# DynGO: a tool for visualizing and mining of Gene Ontology and its associations

**DOI:** 10.1186/1471-2105-6-201

**Published:** 2005-08-09

**Authors:** Hongfang Liu, Zhang-Zhi Hu, Cathy H Wu

**Affiliations:** 1 Department of Information Systems, University of Maryland, Baltimore County, 1000 Hilltop Circle, MD 21050, USA; 2 Department of Biochemistry and Molecular Biology, Georgetown University Medical Center^2^, 3900 Reservoir Road, NW, Washington, DC 20057, USA

## Abstract

**Background:**

A large volume of data and information about genes and gene products has been stored in various molecular biology databases. A major challenge for knowledge discovery using these databases is to identify related genes and gene products in disparate databases. The development of Gene Ontology (GO) as a common vocabulary for annotation allows integrated queries across multiple databases and identification of semantically related genes and gene products (i.e., genes and gene products that have similar GO annotations). Meanwhile, dozens of tools have been developed for browsing, mining or editing GO terms, their hierarchical relationships, or their "associated" genes and gene products (i.e., genes and gene products annotated with GO terms). Tools that allow users to directly search and inspect relations among all GO terms and their associated genes and gene products from multiple databases are needed.

**Results:**

We present a standalone package called DynGO, which provides several advanced functionalities in addition to the standard browsing capability of the official GO browsing tool (AmiGO). DynGO allows users to conduct batch retrieval of GO annotations for a list of genes and gene products, and semantic retrieval of genes and gene products sharing similar GO annotations. The result are shown in an association tree organized according to GO hierarchies and supported with many dynamic display options such as sorting tree nodes or changing orientation of the tree. For GO curators and frequent GO users, DynGO provides fast and convenient access to GO annotation data. DynGO is generally applicable to any data set where the records are annotated with GO terms, as illustrated by two examples.

**Conclusion:**

We have presented a standalone package DynGO that provides functionalities to search and browse GO and its association databases as well as several additional functions such as batch retrieval and semantic retrieval. The complete documentation and software are freely available for download from the website .

## Background

A large amount of data and information about genes and gene products has been stored in various molecular biology databases. To assist with the representation and integration of knowledge about genes and gene products, biological ontologies and tools have been developed. One such ontology is the Gene Ontology (GO) [1], which has become a major vocabulary for annotating genes and gene products in various databases, such as MGI (Mouse Genetic Informatics) [2], SGD (Saccharomyces Genome Database) [3], and RGD (Rat Genome Database) [4], as well as in protein databases such as UniProt (Universal Protein Resource) [5], and InterPro [6]. Genes and gene products from these databases are annotated with GO terms, and GO annotations of genes or gene products are distributed through the GO website as "association files." The databases are referred to as "annotation databases" hereafter.

Annotating genes and gene products is laborious and requires biological expertise – curators need to understand the precise definition and usage of terms as well as their hierarchies in GO for accurate and consistent curation. A browsing tool with the functionality of retrieving descendants and ancestors of a specific GO term will help curators to select the most appropriate GO terms for annotating genes and gene products. Additionally, a tool that shows GO annotations for a list of specified genes and gene products may help the identification of common molecular functions, cellular components or biological processes among these genes and gene products.

**Figure 1:**
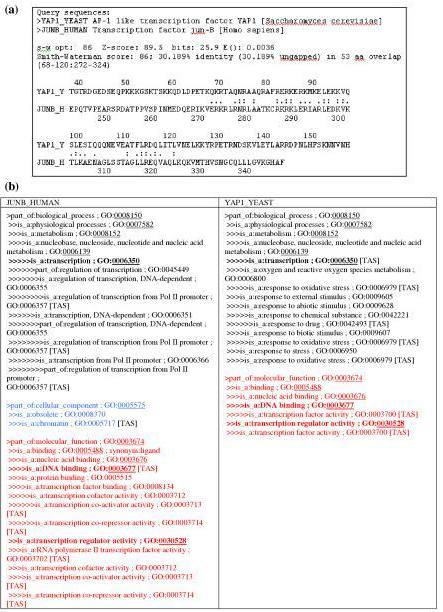
Detection of functional homologs using GO annotations. (a) Sequence alignment based on Smith-Waterman algorithm for YAP1 and JUNB proteins; (b) GO annotations of these two proteins, where the lowest common GO terms in the hierarchies are highlighted based on TAS (Traceable Author Statement) leaf nodes.

Furthermore, the main purpose of having GO is to allow integrated queries across multiple databases. The retrieval of genes and gene products across multiple databases that are semantically related to a query gene or gene product (i.e., genes and gene products that share similar GO annotations with the query gene or gene product) allows users to investigate functional relationships among them. For example, the yeast YAP1 protein (YAP1_YEAST, 650aa) and the human Jun-B protein (JUNB_HUMAN, 347aa) share little sequence similarity (30% sequence identity in 53aa overlapped region) (Figure 1a). However, their functional relationship is revealed by their associated GO annotations, which indicate that both are involved in *transcription *(GO:0006350), *DNA binding *(GO:0003677) and *transcription regulator activity *(GO:0030528). Note that these GO annotations are based on literature citations with the TAS (Traceable Author Statement) evidence code. Such investigation may help biologists to generate new hypotheses.

At the same time, with more genes and gene products having been annotated, many applications have been developed to utilize GO annotations for knowledge discovery. Already over a dozen tools can be found from the GO website for browsing GO terms and GO annotations, and for microarray data analysis. While many tools are available for GO browsing, few allow users to dynamically interact with the retrieved results without losing previous pages; in most web-based tools, activating a link may replace or hide previous pages. A tool that can display GO browsing results in one single window would be helpful to investigate relevant information.

We have developed a tool, DynGO (Dynamic GO), that allows users to dynamically interact with GO annotation data, and to browse all GO terms and annotations. Besides the standard search capability of AmiGO – the "official" GO interface, DynGO provides advanced functionalities such as retrieving and presenting GO annotations based on a list of genes and gene products, or a list of genes and gene products sharing similar GO annotations.

In the following, we describe the graphical user interface (GUI) of DynGO that supports dynamic functionalities of DynGO. Also, we present the semantic similarity of GO, which is used by DynGO for semantic retrieval.

### DynTreeViewer

The GUI of DynGO was adapted from an existing GUI package, DynTreeViewer [7,8]. The viewer, written in JAVA, provides various dynamic tree views for displaying hierarchical data. The program provides functionalities that allow data to be filtered, sorted, deleted, and lifted.

The tree displayed by the DynTreeViewer provides frequency information (or other critical statistical information) for each tree node by combining the frequency counts of all of its child nodes. Various applications using the DynTreeViewer have been developed including a vocabulary development tool based on medical reports [7,8] and a summarization tool for multiple medical reports [9]. Major functionalities of DynTreeViewer that were incorporated into DynGO include tree lifting and tree sorting.

**Figure 2:**
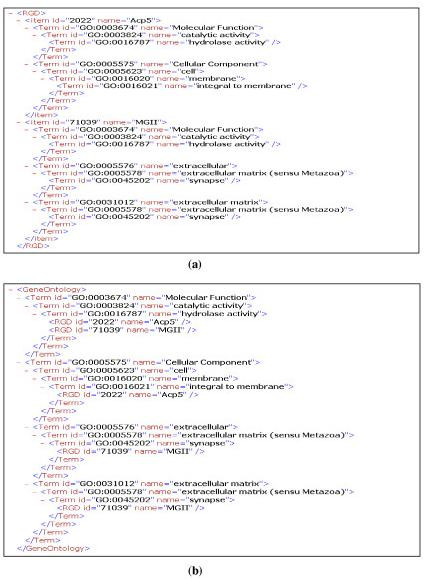
An example of tree lifting. (a) RGD-centric view; (b) GO-centric view.

Tree lifting allows the user to transform the tree and view the data in different orientations dynamically. An example of tree lifting of GO association data in XML format is shown in Figure 2. The data can be displayed based on RGD with GO annotations after lifting the RGD genes and gene products (RGD-centric; Figure 2a) or based on GO hierarchy with the annotation of RGD genes and gene products (GO-centric; Figure 2b). Tree lifting enables users to view hierarchical data through different orientations so that people can switch dynamically from viewing genes and gene products annotated with the same GO term to viewing all GO terms that are associated with the same gene and gene product.

Tree sorting allows the user to view a sub-tree sorted according to the alphabetical (or reversed alphabetical) order, or sorted numerically according to frequency or other statistical information. The sorting functionality allows users to locate nodes easily in a tree.

### GO and semantic similarity of GO terms

GO comprises three hierarchies that hold terms defining the basic concepts of molecular function, biological process, and cellular component, respectively. The terms, organized using a Directed Acyclic Graph (DAG), may have one or more parents with a relation type of either "*is-a*" or "*part-of*." GO terms are used to annotate entities in dozens of genomic and protein databases. The development of GO and its wide adoption for genome annotation makes it feasible to perform semantic retrieval from multiple databases (i.e., to retrieve entities with similar GO annotations as the query entity).

As with any other similarity search task, semantic retrieval requires the definition of a similarity measure for entities sharing similar GO annotations. In DynGO, we adapted one of the semantic similarity measures reported by Lord et al. [10], which is based on the notion of "information content" – i.e., a term in an ontology is more informative if the term and its descendants have fewer annotated genes or proteins. The GO annotation measure starts with a probability measure of each term *t*. Let *D*_*t *_be the collection of GO terms that are either *t *or its descendants Let *A*(*t*, *c*) be the occurrence of *t *annotations given a collection *c*. The probability of *t *in *c*, or *p*(*t*, *c*), is defined as:



Let *CA*(*t*_1_, *t*_2_) be the lowest common ancestor set for terms *t*_1 _and *t*_2_, since GO allows multiple parents for each term. The semantic similarity of two GO terms is defined as:



The similarity of two genes or gene products is then defined as the average similarity between GO annotations for them.

## Implementation

**Figure 3:**
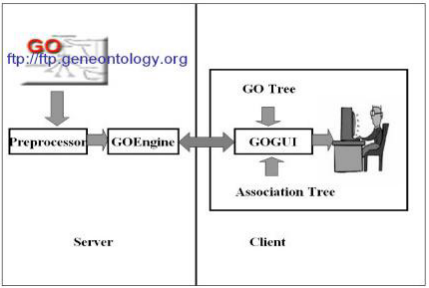
The overall server-client architecture of DynGO.

DynGO was designed as a server-client application. As depicted in Figure 3, DynGO contains three functional components: *Preprocessor *and *GOEngine *running on the server side [see Additional file 1], and *GOGUI *on the client side [see Additional file 2]. The Preprocessor uses the GO distribution file as input and generates several database tables, while the GOEngine accepts queries from the client and generates trees for displaying in GOGUI.

### Server – preprocessor and GOEngine

Both the preprocessor and GOEngine were coded using PERL, an open source programming language. The Preprocessor dynamically downloads the GO distribution file go_YYYYMM-assocdb.rdf-xml.gz (YYYY stands for year and MM stands for month) from the GO ftp site and generates tables that are stored in BerkeleyDB, an open source database management system. Information stored in the database includes hierarchical relations, GO terms and attributes (e.g., names, synonyms, references, and definitions), and GO annotations and attributes (e.g., names and references) of genes and gene products. Some intermediate tables for semantic retrieval are also generated and stored, including the probability table of terms in collections shown in Formula 1 and the semantic similarity table storing the similarity of any pair of GO terms computed according to Formula 2. Note that the collection used to derive the probability of GO terms consists of all entries from annotation databases in the GO distribution file.

The GOEngine processes queries and generates trees. Two types of trees can be generated depending on the nature of the queries: a GO tree or an association tree. The GO tree arranges GO terms according to the GO hierarchies. The association tree arranges genes and gene products as leaf nodes of associated GO terms according to the GO hierarchies. In the current implementation, the GOEngine supports seven types of queries as listed below, with the function name followed by its argument(s) in parentheses.

• **Generate_GO () **generates a GO tree consisting of all GO terms where a term may appear in multiple branches of the tree.

• **Generate_Assoc (AssocDB) **creates an association tree for all genes and gene products in a given association database, AssocDB (e.g., MGI). GO terms not associated with genes and gene products in AssocDB are not shown in the tree. A gene entity may appear in multiple branches of the tree if it is annotated by multiple GO terms.

• **Retrieve_Genes (GeneIDs) **creates an association tree for all genes and gene products from a list of GeneIDs in one or more annotation databases, where each GeneID is the unique identifier used in the corresponding association database (e.g., MGI:108111).

• **Retrieve_Relatives (GeneID, AssocDBs, Parameters) **retrieves genes and gene products that are "relatives" of the GeneID based on similar GO annotations from a list of annotation databases (AssocDBs), and displays the related genes and gene products in an association tree. Several parameters are needed for the function: parameters that assign weight to each hierarchy (the default values are 0.4 for molecular function, 0.4 for biological process, and 0.2 for cellular components) and the similarity threshold value (the default value is 0.5).

• **Retrieve_Genes (GoTerm, AssocDBs) **returns genes and gene products from a list of annotation databases (AssocDBs) for a query GO term (GoTerm), and displays the entities in an association tree.

• **Retrieve_Descendants (GoTerm) **generates a GO tree consisting of all ancestors and descendants of a given GoTerm.

• **Retrieve_Search (QueryString, AssocDBs) **searches IDs, terms, and attributes of all GO terms or genes and gene products in AssocDBs using a query string that can be a word or any identifier (such as GO identifiers or gene entity references). All matches found from GO or AssocDBs are displayed in a GO tree or an association tree.

### Client – GOGUI

**Figure 4:**
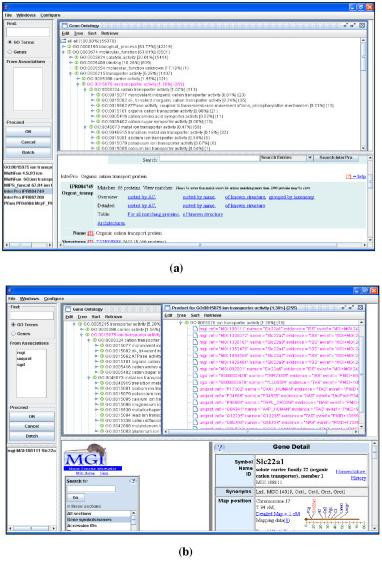
Screen shots of DynGO. (a) GO tree obtained from all GO terms; (b) Association tree obtained by retrieving products for GO:0015075.

The JAVA-based client interacts with users using menus, mouse clicks, or user input dialogs. The primary interface of GOGUI is a four-panel window in which the user can inspect the GO hierarchies and GO annotations. Figure 4a shows a screenshot after loading the GO tree generated by the function *Generate_GO*. The left-top panel of the window (InputPanel) handles user queries to GO and GO annotation databases. The right-top panel (TreeHolder) displays trees. The tree shown in Figure 4a has been sorted using probability information. The left-bottom panel (ReferencePanel) lists all database cross-references for the selected tree node. The right-bottom panel (WebPanel) displays the website of the selected tree node or the selected references. The example in Figure 4a shows that, after choosing the GO node "*GO:0015075 ion transporter activity*," the reference list for GO:0015075 was displayed in the ReferencePanel; and that the selection of "InterPro IPR004749" from the reference list returned the website of the InterPro reference in the WebPanel.

The TreeHolder can hold multiple trees, where each tree is displayed using DynTreeViewer. Figure 4b displays two trees, the GO tree and an association tree. The latter is obtained by the function *Retrieve_Genes*, where genes and gene products for GO term *ion transporter activity *were retrieved from three associated databases MGI, UniProt, and SGD. Other panels in GOGUI are also dynamically changed to indicate the current tree. The MGI website of a selected gene "MGI:108111" is shown at the WebPanel. Links shown in the website can also be activated.

**Figure 5:**
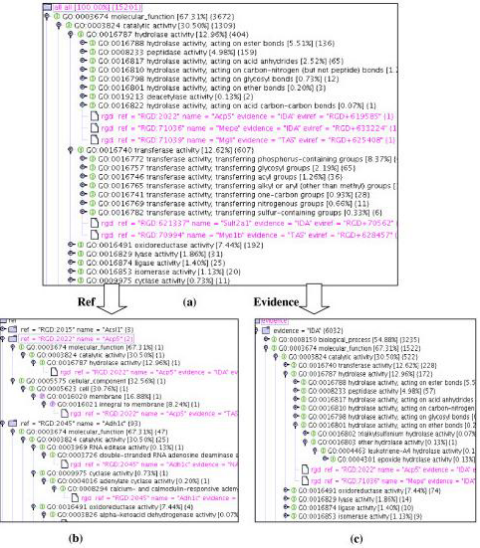
Tree lifting function to dynamically obtain trees with different orientations. (a) Association tree for RGD; (b) View obtained by lifting reference identifiers; (c) View obtained by lifting evidence codes.

**Figure 6:**
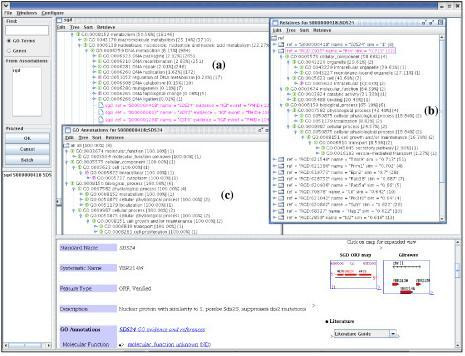
Screen shot for exploring association trees. (a) Association tree for all genes in SGD; (b) Relatives of gene SDS24; (c) Annotations for SDS24.

Figures 5 and 6 illustrate the flexibility of various tree display options in GOGUI and the functionality of semantic retrieval. Figure 5 shows three orientations of an association tree for RGD generated by the function *Generate_Assoc (RGD)*: the default association tree which arranges RGD genes and gene products as leaf nodes of GO terms, where users can easily identify entities associated with a specific GO term (Figure 5a); the "lifted" tree arranged based on RGD entities, where users can view GO annotations for a specific gene or gene product (Figure 5b); and the tree that arranges annotations according to their evidence codes (Figure 5c). Figure 6 shows the functionality of semantic retrieval using three association trees: the default association tree that displays the GO annotations for SGD generated by the function *Generate_Assoc (SGD) *(Figure 6a); the tree that shows the annotations for one gene, SDS24 (Figure 6b); and the tree that displays the semantically related genes and gene products for gene SDS24 sorted according to the semantic similarity (Figure 6c). The latter was obtained dynamically using the function *Retrieve_Relatives *with default parameters to identify entities with similar GO annotations to those of SDS24 from RGD. As indicated in Figure 6c, the closest related gene in RGD to yeast gene SDS24 in SGD is the rat gene Itsn. By overlaying Figures 6b and 6c, which show the detailed annotations for Itsn and SDS24, respectively, users can compare their GO annotations – for example, both genes are associated with *GO:0006810 transport*.

## System testing and example applications

We conducted an integrated system testing and analysis using the November 1, 2004 GO distribution file go_200411-assocdb.xml.gz [11]. There were 18,017 GO terms in this distribution, which were used to annotate 1,004,671 genes and gene products from 17 different annotation databases. It took about 5 hours for the Preprocessor to prepare all database tables on a PC laptop running Window XP with a 1.6 GHz Intel processor and 512 MB of RAM. One of the most computing-intensive tasks is the construction of the similarity table, where the similarity of every pair of GO terms is computed and stored. Note that semantic retrieval operation is a kind of time-consuming when the number of records in the association database is over ten thousands. For users who do not have plans to use the semantic retrieval function of DynGO, a light version of the Preprocessor is available that can prepare tables much faster (i.e., in less than 10 minutes using the same laptop).

The basic searching and retrieving functionality of DynGO was tested by four users (bioinformatics researchers and PhD students). They were pleased that DynGO could **instantly **retrieve the GO hierarchy for a specific GO term and retrieve a GO annotation tree for a specific gene or gene product. It was commented that DynGO is easy to move around to gather information about GO terms as well as GO annotations for a specific gene or gene product since multiple views were held in one window. Some indicated that semantic retrieval was slow (longer than 5 minutes) when they used annotation databases with more than thousands of records.

Besides searching and browsing GO terms and GO annotation databases, the advanced functionalities of DynGO allow it to be used for applications where records are annotated using GO. Here, we illustrate through two applications: one is to visualize GO annotations of informative probe sets for microarray data analysis [12] and the other is in the study of complementing GO with PIRSF classification-based protein ontology [13].

**Figure 7:**
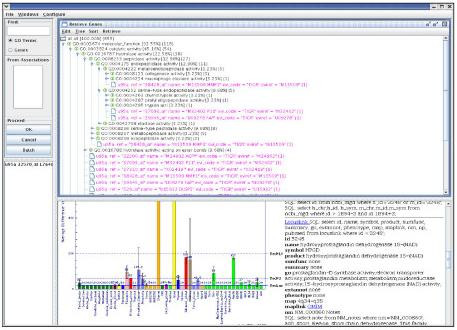
DynGO for the visualization of annotated probe sets for microarray data analysis.

The example data set we used in visualizing GO annotations of informative probe sets is the Head and Neck Squamous Cell Carcinoma (HNSCC) data set, in which differentially expressed genes in head and neck cancer were examined by Kuriakose et al [12]. In the study, RNA extracted from 22 paired samples of HNSCC and normal tissue from the same donors was hybridized to the Affymetrix U95A chip. Forty-two differentially expressed probe sets were selected for further validation by hierarchical clustering, multiple probe-set concordance, target-subunit agreement, and real-time PCR analysis. We downloaded the GO annotations for the probe sets of the U95A chip from the TIGR Resource website [14] and treated them as an association database. In DynGO, we used the function *Retrieve_Genes (GeneIDs)*, where elements in GeneIDs are annotated probe sets. Users could then investigate the GO annotations in multiple orientations, inspect different statistical measures such as probability of a GO term in the set, retrieve genes that hold similar annotations from other annotation databases, among other functions (Figure 7).

**Figure 8:**
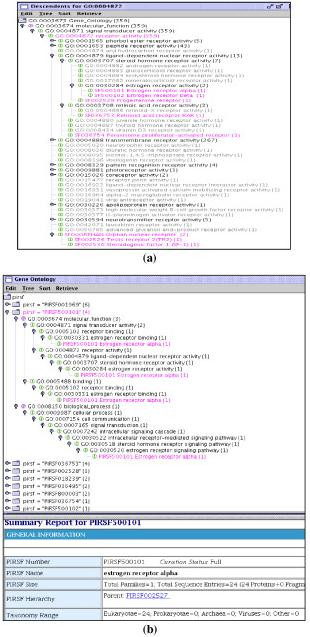
DynGO for the study of complementing GO with the PIRSF protein classification system. (a) Identification of missing GO nodes according to PIRSF families; (b) Linkage of GO sub-ontologies based on PIRSF GO associations.

The study of GO and PIRSF [15] mapping shows that PIRSF classification-based protein ontology can complement GO concepts by identifying missing GO branches/nodes and linking GO terms among the three sub-ontologies (i.e. molecular function, biological process, and cellular component) (Figure 8). In the study, we mapped PIRSF protein families to the GO hierarchy and used DynGO to superimposing the two classification hierarchies with a bidirectional display showing either a GO-centric (Figure 8a) or a PIRSF-centric (Figure 8b) view to explore GO and PIRSF relationships.

We found that the majority of curated PIRSF families map to GO leaf nodes, many of which also share common GO leaf nodes. The PIRSF associations to GO nodes allow us to examine whether certain GO subtrees might need expansion if GO concepts are too broad and to identify missing GO nodes when entire groups of superfamilies cannot be mapped to existing GO terms. As an example, Figure 8a shows that nested under *receptor activity *(GO:0004872) is a leaf node *estrogen receptor activity *(GO:0030284), under which we can identify two distinctly different concepts estrogen receptor α and β (SF500101, SF500102) that are not represented in GO. Also identified are activities of several groups of gene products missed in GO: progesterone receptor (SF002528) of *steroid hormone receptor activity *(GO:0003707), and retinoid acid receptor RAR (SF036753) of *retinoid acid receptor activity *(GO:0003708). Furthermore, directly under *receptor activity *there is no concept for orphan nuclear receptor (ligand-independent nuclear receptor) activity, which encompasses a large group of gene products such as TR2 (SF002526) and SF-1 (SF002530).

PIRSF classification can also provide links between the three GO sub-ontologies, each of which presently has its own hierarchical organization with no relationships inter-connecting them. Figure 8b shows the PIRSF-centric view where estrogen receptor α (PIRSF500101) is annotated with GO terms in both molecular function and biological process, which connects the *receptor binding *(GO:0005102) of *signal transducer activity *(GO:0004871) with the *steroid hormone receptor signaling pathway *(GO:0030518) of *signal transduction *(GO:0007165).

## Discussion

In this paper, we adapted one of the similarity measures used by Lord et al [10] for semantic retrieval. We plan to investigate other similarity measures for GO terms (e.g., the tree similarity measure used by Wang and Zhao [16]) and incorporate them into DynGO. Note that we include the evidence code when displaying the trees. Researchers that wish to filter out the annotations whose evidence codes are IEA can lift the tree according to the evidence code so that IEA annotations can be deleted from the tree.

Many software tools have been developed for GO, most of which are web-based browsing systems. Some of these web-based systems do not provide tree navigation where all GO terms are loaded; instead, the browsing is limited only to queries of a single GO term as in GenNav [17]. Other systems provide navigation function for all GO terms but do not accept queries, such as the GO Browser developed at NCI's Cancer Genome Anatomy Project. A few tools do accept queries and have full tree navigation, such as the MGI GO Browser [18], but the results are shown in flat-tables so that users cannot directly inspect relations among multiple GO terms or genes and gene products. Additionally, all such web-based systems depend on Internet connection and may experience slow response time.

There is one standalone tool that is similar to DynGO, the Berkeley Drosophila Genome Project (BDGP) DAG-EDIT [1]. Both tools are JAVA-based and display search results in tree views. DAG-EDIT, however, is designed as an editor for GO term editing; therefore, it does not provide the functionality for browsing genes and gene products in association trees and for dynamically linking to the websites. DynGO, on the other hand, is for querying, visualizing, and mining GO terms as well as GO annotations from annotation databases. DynGO enables GO curators and users to load multiple trees, and to dynamically obtain views in different orientations for the resulting trees.

Note that few of the existing tools support semantic retrieval (i.e., to retrieve genes and gene products sharing similar annotations), batch retrieval (i.e., to retrieve GO annotations for a list of entities), or allow users to dynamically display trees with different orientations at the same time. These features distinguish DynGO from other tools.

Several GO mining tools are available. These include GoMiner [19], MAPPFinder [20], FatiGO [21], and GoSurfer [22]. Zeeberg et al. [19] did an extensive comparison of GoMiner with some of these mining tools. Here, we concentrate on comparing GoMiner and DynGO. GoMiner is a program package that organizes a list of interesting genes for biological interpretation using GO. It includes statistical analysis and two visualizations: a hierarchical tree view and a DAG view. While DynGO does not perform advanced statistical analysis, it can display the GO annotation trees for a gene list identified and exported from other software. GO terms for clusters of differentially expressed genes can be easily detected using the statistical information provided in the tree, either from the leaf descendant statistics or from the microarray data analysis software. Instead of providing two views as in GOMiner, we present the result in one tree that can be manipulated dynamically to change the orientation or to display information such as definitions or associated websites of a node. DynGO is flexible in the sense that it can be coupled with any microarray data analysis software to display the GO annotation for informative genes. Meanwhile, it combines well with browsing functionality and allows users to view the overall GO tree structure.

In the future, we will extend DynGO to incorporate microarray data analysis software on the server side to allow advanced microarray data analysis. Additionally, we plan to improve DynGO by collecting comments and feedback from the GO community. A formal usability evaluation is also planned.

## Conclusion

We have presented a standalone package DynGO that provides functionalities to search and browse GO and its association databases as well as several additional functions such as batch retrieval and semantic retrieval. It enables users to browse the whole GO as well as GO annotations for many genomics and protein databases. It also includes statistical measures such as the number of leaf nodes and the probability of a specific GO term being assigned for a given collection. The results are displayed as trees that can be sorted; or users can dynamically obtain views with different orientations. For GO curators, curators of genomic and protein annotation databases, and users who use GO frequently, DynGO provides fast and convenient access to the GO and gene entity data since the data are located locally (the same computer or a computer on a local intranet) and the DynGO interface holds all results inside one browsing window. DynGO can also be extended to view any other data sets where the GO annotations are available.

## Availability and requirements

• Project name: DynGO

• Project homepage: 

• Operating systems: platform independent, tested on Window XP system and Red Hat Linux System

• Programming language: Perl, Java

• Other requirements: Java version 1.4, Perl version 5.8, Berkeley DB, Perl model BerkeleyDB

• License: GNU GPL

• Any restriction to use by non-academics: license needed.

Documentation, the source code of the server, and the executable of the client are available in the project website [also see Additional files]. Other used software components are available at the according sources.

## Supplementary Material

Additional file 1 client_download.zip The executable jar file for client Click here for file

Additional file 2 server_download.zip The source code for server Click here for file

Additional file 3 dyngo.html The documentation for DynGO Click here for file

Additional file 4 dingo-download.html The tutorial for downloading DynGO Click here for file
