# Supplementary material for: DynGO: a tool for visualizing and mining of Gene Ontology and its associations
Source: BMC Bioinformatics. 2005 Aug 9;6:201. doi: 10.1186/1471-2105-6-201 (PMC1199584; doi:10.1186/1471-2105-6-201)
Supplement: Additional file 3 — dyngo.html The documentation for DynGO [file 1471-2105-6-201-S3.html]

DYNGO: A Tool for Browsing and Mining Gene Ontology and Its Associations 


**DYNGO: A Tool for Browsing and Mining Gene Ontology and Its Associations**

#### People: Dr. Hongfang Liu , Dr. Zhangzhi Hu , Dr. Cathy Wu

---

About | Implementation Support | Download | Publications | | |


---


### About

DYNGO is a standalone package that provides similar browsing functionality to the official GO browsing tool,  AmiGO. It is extended from a tree browser called dynTreeViewer. DYNGO also allows users to load a list of entities and retrieve the corresponding GO annotations. It enables users to retrieve gene or gene products that hold similar annotations. The retrieved result is shown in a tree organized according to GO hierarchies, and the tree can be mainpulated dynamically by sorting and changing orientation. DYNGO can aslo be used for MicroArray data analysis using GO annotations and for other applications.

### Implementation

DYNGO was designed as a server-client application. Figure 1 depicts an overview of DYNGO. It contains three functional components: Preprocessor, GOEngine, and GOGUI. The Preprocessor and Tree Generator run on the server side while GOGUI runs on the client side. On the server side, the input to the Preprocessor is the GO distribution, and several tables were then generated and stored in a database. The Preprocessor can dynamically get the distribution from the GO ftp site. The GOEngine takes queries from the client side and generates trees for displaying in GOGUI.

- #### Preprocessor and GO Engine

  Both the Preprocessor and GOEngine were coded using PERL, an open source programming language. The Preprocessor downloads the GO distribution and generates tables which are stored in a database using an open source database management system, BerkeleyDB. Information stored in the database includes hierarchy relations, GO term properties (e.g., names, synonyms, references and definitions), GO annotations for genetic entities and their names and references, and several intermediate tables which store data for semantic retrieval.
  The GOEngine processes queries and generates trees. Two types of trees can be generated depending on the nature of the queries. One is a GO tree which arranges GO terms according to the GO hierarchies. The other type is an association tree which arranges gene products as leaf nodes of associated GO terms according to the GO hierarchies. In the current implementation, there are seven types of queries that the GOEngine can handle.
  - #### Generate\_GO ()

    it generates a GO tree including all GO terms. Note that one term may appear in multiple branches of the tree.- #### Generate\_Assoc (AssocDB)

      it creates an association tree for a given association database AssocDB (e.g., Mouse Genetic Informatics, MGI [7]). GO terms which do not have gene products from AssocDB as descendents are absent in the tree. Similar to the tree generated by Generate\_GO, one gene product may appear in multiple branches of the tree if it is annotated by multiple GO terms.- #### Retrieve\_Genes (GeneIDs)

        it creates an association tree for gene products from a list of GeneIDs where each element in GeneIDs is the identifier used in the corresponding association database (e.g., MGI:108111) and the list can be from different association databases.- #### Retrieve\_Relatives (GeneID, AssocDBs, Parameters)

          it retrieves gene products from a list of association databases AssocDBs which have similar GO annotations as GO annotations of GeneID. The operation is an expensive operation- #### Retrieve\_Products (GoTerm, AssocDBs)

            it returns gene products from a list of association databases AssocDBs for a query GO term GoTerm. The gene products are displayed as an association tree.- #### Retrieve\_Search (QueryString, AssocDBs)

              the query string here can be a word or any identifiers such as GO term identifier or Gene Product references. It returns all matches found from GO or AssocDBs in a GO tree or an association tree.- #### Client - GOGUI

    The client was coded using JAVA and it interacts with users using menus, mouse clicks, or user input dialogs. The primary interface of GOGUI takes a four-panel window in which the user can inspect the GO hierarchies and GO annotations. Figure 2 shows a screenshot after loading the tree generated by calling the function Generate\_GO(). The panel at the left-top corner of the window handles user queries to GO and GO association databases. The panel at the right-top corner of the window is called TreeHolder; it displays trees. The tree shown in Figure 3  has been sorted using probability information. The bottom half of the window includes two panels. The left-bottom panel lists references of the current selected tree node. The right-bottom panel displays the website of the selected tree node or its references when choosing from the list in the left-bottom panel. For example, after choosing GO:0015075 (i.e, ion transporter activity), a list containing the references for GO:0015075 was displayed in the left-bottom panel. Then selecting a reference (i.e., InterPro IPR004749) from the list, the website of the reference was displayed in the panel at the right-bottom corner.

    TreeHolder can hold multiple trees where each tree is displayed using DynTreeViewer which was extended to include some additional functions. One function added for a GO tree is retrieving genetic entities associated with a selected GO term. Figure 3 displays an association tree obtained by retrieving the entities for GO term ion transporter activity from three associated databases MGI, UniProt, and Saccharomyces Genome Database (SGD). Other panels in GOGUI are also dynamically changed to indicate the current tree. The MGI website of a selected genetic entity MGI:108111 is shown at the right-bottom panel. Links shown in the website can also be activated. Another additional function for a GO tree is the retrieval of descendents for a selected GO term. For an association tree, the function of finding gene products with similar annotations (i.e., semantic retrieval) was added. Figures 4 and 5 illustrate the flexibility of visualizing trees displayed in GOGUI and the functionality of semantic retrieval. In Figure 4, three orientations of an association tree for Rat Genome Database (RGD) are shown: the top one is the original association tree which arranges RGD entities as leaf nodes of GO terms where users can easily identify entities that are associated with a specific GO term; the tree at the left-bottom arranges these entities as the children of the root where users can easily check GO annotations for a specific entity; and the tree at the right-bottom arranges entities according to their evidence codes. The function of finding relatives allows users to inspect entities with similar GO annotations from multiple association databases. Figure 5 shows three association trees which were obtained dynamically: the tree at the top displays the GO annotations for SGD; the tree at the left-bottom shows the annotations for one gene, SDS24; and the tree at the right bottom displays the relatives for gene SDS24 which are sorted according to the similarity. The similarity measure of two entities was computed using the average weighted similarity measure of GO terms that are associated with them, assigning the default weights to the three GO aspects. From Figure 5, we can see that rat gene Dnm2 is closest to gene SDS24 in SGD. Users can inspect GO annotations of these two genes by checking their annotations. The tree at the right side in Figure 5 shows the detailed annotation for Dnm2, and the tree at the left bottom shows the annotation for SDS24. From the figure, we can see that both entities are associated with GO:0006897 (i.e., endocytosis).

### Support

DYNGO was developed using JAVA 1.4 , PERL 5.8  and  BerkeleyDB 4.2.52 . In order to install and run DYNGO, your system should have the latest version of JAVA, PERL and BerkeleyDB installed.

### Download

Follow the instruction provided in the README file included in the package for installation and execution of the project.

Download the source codes

### Publications
