# Supplementary material for: DynGO: a tool for visualizing and mining of Gene Ontology and its associations
Source: BMC Bioinformatics. 2005 Aug 9;6:201. doi: 10.1186/1471-2105-6-201 (PMC1199584; doi:10.1186/1471-2105-6-201)
Supplement: Additional file 4 — dingo-download.html The tutorial for downloading DynGO [file 1471-2105-6-201-S4.html]

## DYNGO: *Dyn*GO *Dyn*amically browsing *G*ene *O*ntology (Download and Tutorial)

## Download and Install

DYNGO was developed using JAVA 1.4 , PERL 5.8  and  BerkeleyDB 4.2.52 . In order to install and run DYNGO, your system should have the latest version of JAVA, PERL and BerkeleyDB installed.

1. **Download Server**  Download the DYNGO\_server program  here  to computers that will serve as the server. Extract the files in the zip file to a directory. In this directory,
   there are three subdirectories (data, server, result) and a README file.
2. **Download Gene Ontology** Go to the subdirectory data, download the latest version of Gene Ontology  Download GO
3. **Set up Server**  Two versions of servers included. One is without semantic retrieval function (~ 10 minutes) and the other includes semantic retrieval function (~ 4 hours execution). Check README in the directory on how to set up each
4. **Start Server**  You can start server by going to server directory, and type "perl dyngo\_server.pl"
5. **Download Client**  Download the DYNGO\_client program  here  to computers that will be used by users to explore GO and its associations. Extract the files to a directory. In this directory, type command "java -jar dyngo.jar" or "start\_client"

## Short Tutorial

### Configure Menu

After starting DYNGO client, a window will pop up. By default, the client will assume that the server is located in local machine. To configure the client to the active server, go to the configure menu and set up the server.

### File Menu

1. **Load GO** Load Gene Ontology
2. **Load Assoc** Load a selected association database with Gene Ontology annotation
3. **Save** Save the tree shown in the top frame
4. **Load Genes** Load GO annotations for a list of genes included in a file  Sample File for a list of genes

### Search Panel

It is similar to AMIGO searching function. You can search for GO terms or Gene Products

### DynTreeViewer Menu

1. **Edit**  Allow users to delete a node and its descendents
2. **Tree**  Allow users to expand or collapse the tree by one, two, or all levels. It also allows the user to transform the tree to dynamically change the orientation
3. **Sort**  Allow users to view the branch sorted by different kinds of orders
4. **Retrieve**  Allow users to retrieve GO annotations or Gene Products, Decendents among others
